# Tailoring Crystal Growth Regulation and Dual Passivation for Air‐Processed Efficient Perovskite Solar Cells

**DOI:** 10.1002/advs.202407401

**Published:** 2025-02-19

**Authors:** Qianyi Li, Dongyang Li, Zhiqi Li, Qiong Liang, Patrick W. K. Fong, Yu Han, Kuan Liu, Jiangsheng Yu, Peng Bai, Tao Zhu, Yang Bai, Guang Yang, Zhiwei Ren, Gang Li

**Affiliations:** ^1^ Department of Electrical and Electronic Engineering Photonic Research Institute (PRI) Research Institute of Smart Energy (RISE) The Hong Kong Polytechnic University Hung Hom, Kowloon Hong Kong 999077 China; ^2^ Research Institute for Intelligent Wearable Systems (RI‐WEAR) The Hong Kong Polytechnic University Hung Hom, Kowloon Hong Kong 999077 China; ^3^ Faculty of Materials Science and Energy Engineering Shenzhen University of Advanced Technology Shenzhen 518107 China; ^4^ Institute of Technology for Carbon Neutrality Shenzhen Institute of Advanced Technology Chinese Academy of Sciences Shenzhen 518055 China

**Keywords:** 3‐Guanidinopropionic acid, air processed, dual engineering strategy, perovskite solar cells

## Abstract

Hybrid metal halide perovskite solar cells (PSCs) are emerging as highly competitive next‐generation photovoltaics due to their excellent performance and low production cost. However, the construction of high‐efficiency PSCs typically requires an inert nitrogen environment within a glove box, inadvertently increasing manufacturing costs and hindering the transition from lab‐scale to industrial‐scale production. In this work, an air ambient fabrication of pure α‐phase FAPbI_3_ PSCs with high‐efficiency and stability, utilizing a dual‐functional engineering strategy assisted by 3‐Guanidinopropionicacid (3‐GuA) is reported. 3‐GuA assists in managing excess PbI_2_ and promotes the formation of high‐quality FAPbI_3_ films via intermolecular exchange. Simultaneously, the existence of 3‐GuA minimizes the defects and stabilizes the resulting perovskite films. As a result, the ambient‐air fabricated PSCs achieve a power conversion efficiency (PCE) of 24.2% with negligible hysteresis and excellent stability. Additionally, these devices demonstrate superior reproducibility, offering valuable guidance for future advancements in this technology.

## Graphical abstract

In this work, a straightforward dual functional strategy, assisted by 3‐GuA, is demonstrated to achieve air ambient fabricated FAPbI_3_ PSCs with excellent efficiency and stability. Systematic investigations indicate that 3‐GuA aids in the management of excess PbI_2_ and facilitates high‐quality perovskite film formation. Furthermore, 3‐GuA predominantly interacts with the grain boundaries and surfaces of the perovskite films, serving to passivate defects and stabilize the crystal structure. Consequently, devices with 3‐GuA demonstrate superior reproducibility, providing valuable roadmap for future advancements in air ambient industrial fabrication.

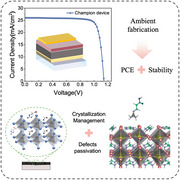

## 1 Introduction

Organic–inorganic hybrid perovskite has emerged as a tremendous potential photovoltaic material due to its high efficiency and low material cost.^[^ ^1^ ^]^ Thanks to massive efforts in materials innovation and structure design, the certified power conversion efficiency (PCE) of perovskite solar cells (PSCs) has considerably improved from 14.1% to 26.1%.^[^ ^2^ ^]^ Importantly, PSCs can be produced through a solution‐based fabrication process, making them compatible with industrial‐scale production.^[^ ^3^ ^]^ The high‐performing PSCs show great promise in potentially rivaling multi‐crystalline Si photovoltaics. However, the fabrication of these high‐efficiency and stable PSCs is typically confined to a restricted fabrication condition, usually in an inert atmosphere within nitrogen/argon glovebox.^[^ ^4^ ^]^ Clearly, maintaining such severely restricted fabrication conditions unintentionally escalates manufacturing costs and poses a barrier to the transition from lab‐scale to industrial‐scale production.^[^ ^5^ ^]^ Therefore, it is highly desirable to develop high‐efficiency PSCs under ambient atmosphere condition.^[^ ^6^ ^]^ To tackle this problem, various approaches have been developed to fabricate high‐efficiency PSCs without the need for the inert atmosphere, for example, a thermal radiation protocol, the solvent dripping method, and the additive strategy.^[^ ^7^ ^]^ By contrast, the two‐step fabrication method possesses the advantages of environmentally friendly fabrication, easy processing, and excellent repeatability, especially fabrication in ambient condition.^[^ ^4^, ^8^ ^]^ Remarkably, the uncontrollable stoichiometric ratio and unreactive conversion between inorganic components and organic components are still the big challenge for PSCs based on two‐step formation.^[^ ^9^ ^]^ It is desirable to develop viable two‐step strategies to fabricate high‐efficiency PSCs in ambient air. An essential challenge in air‐based perovskite preparation is the susceptibility of perovskite precursors and the final material to moisture. This necessitated the exploration of new material compositions and processing techniques in air‐based fabrication processes for perovskite. Researchers have investigated various components to enhance the durability of perovskite films against moisture and oxygen exposure. Through iterative development and optimization, these materials and methods have continuously advanced the efficiency and stability of perovskite solar cells. Notably, materials containing the guanidine functional group have demonstrated passivation capabilities, yielding relatively high‐PCEs. The combination of 3‐Guanidinopropionicacid (3‐GuA) advancements and the all‐air fabrication approach resulted in significant enhancements in perovskite solar cell performance. The ambient fabrication simplifies manufacturing processes, making conventional equipment and facilities viable. This compatibility with current manufacturing infrastructure eases the integration of perovskite technology into industrial workflows, thus promoting rapid adoption and scalability. Additionally, the ability to produce high‐quality perovskite films in ambient conditions supports flexible and distributed manufacturing models, potentially enabling localized production and reducing the carbon footprint associated with material and device transportation. The successful development of techniques for producing high‐efficiency perovskite solar cells in air using the two‐step method offers numerous benefits, including cost‐effective, scalable manufacturing and so on. Our future research efforts will concentrate on optimizing air‐based fabrication methods and exploring new material systems to fully exploit the potential of perovskite solar technology.

In this work, we demonstrate a facile 3‐GuA assisted dual‐functional strategy to facilitate two‐step fabrication of high‐quality FAPbI_3_ PSCs, independent of ambient atmospheric condition. Systematic investigations illuminate that the incorporation of 3‐GuA promotes the formation of high‐quality FAPbI_3_ film via self‐forming porous PbI_2_ microstructure and subsequent intermolecular exchange. Meanwhile, multifunctional 3‐GuA effectively anchors on the surface of perovskite crystals, thereby reducing defects and stabilizing the resultant perovskite films. The optimized PSCs achieved a short‐circuit current density (*J_SC_*) of 25.27 mA cm^−2^, a Fill Factor (FF) of 81.68%, and an open‐circuit voltage (*V_OC_*) of 1.17 V, giving rise to a champion PCE of 24.2% without hysteresis. In addition, the stability of PSCs device is significantly improved, maintaining 93% of its initial PCE after 960 h without further encapsulation.

## 2 Results and Discussion

**Figure 1:**
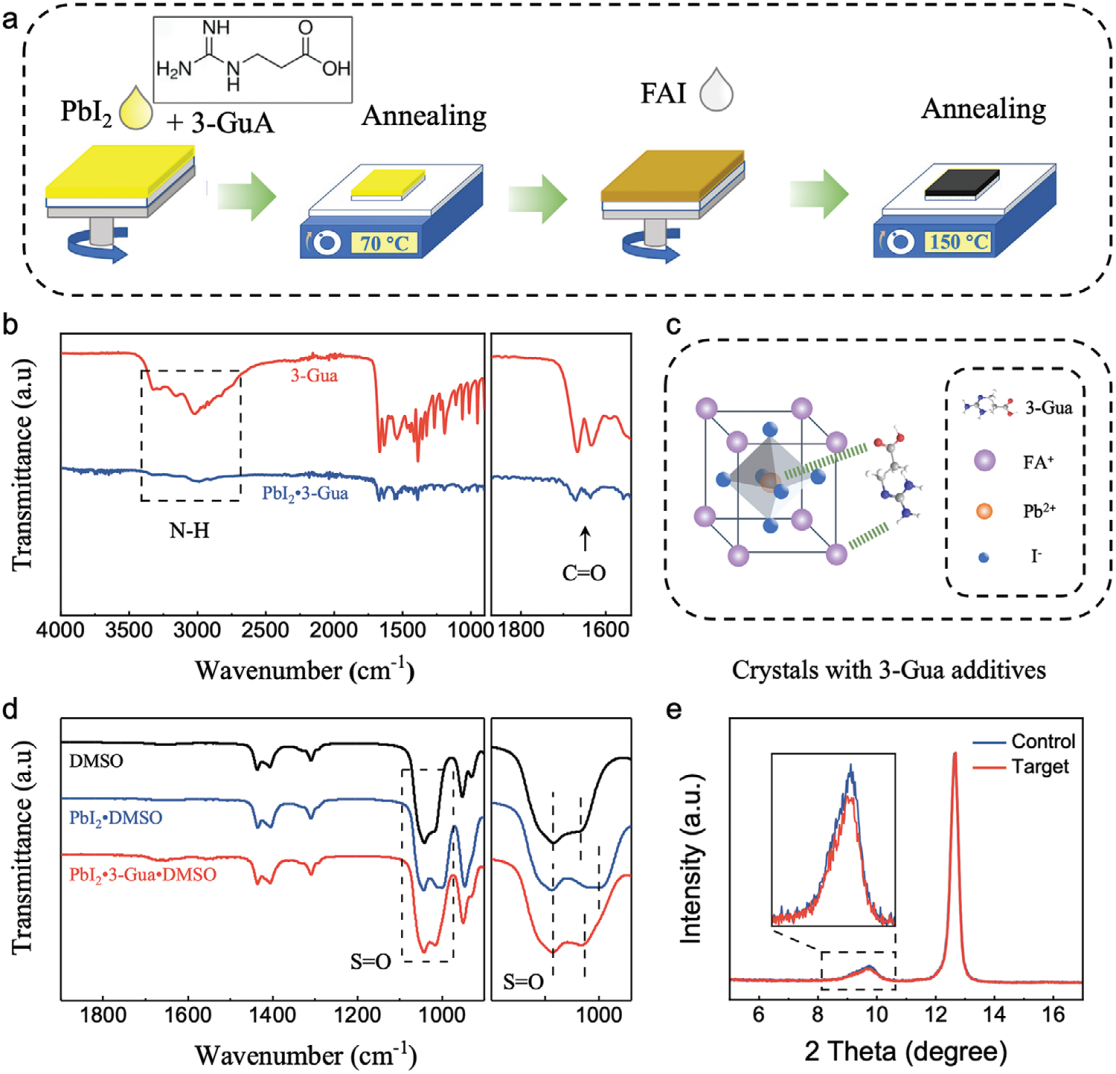
Device fabrication and film characteristics. a) Schematic diagram of the spin coating process. b) Fourier transform infrared (FT‐IR) spectra of 3‐GuA and PbI_2_·3‐GuA powder. c) Schematic diagram of the interaction between 3‐GuA and FAPbI_3_ perovskite. d) Fourier transform infrared (FT‐IR) spectra of the DMSO solvent, PbI_2_ dissolved in DMSO, and PbI_2_·3‐GuA dissolved in DMSO. e) X‐ray diffraction (XRD) patterns of the control and target PbI_2_ films.

The FAPbI_3_ perovskite films were prepared using a two‐step fabrication process, which is shown in **Figure** **1**a. The 3‐Guanidinopropionicacid (3‐GuA) in Figure 1a (inset) was incorporated into PbI_2_ precursor in the first step. More details can be found in the Experimental Section. Fourier transform infrared (FT‐IR) spectroscopy was first applied to analyze the interaction between 3‐GuA and PbI_2_. As shown in Figure 1b, the stretching vibrations at wavenumbers of 1670 cm^−1^ and 1632 cm^−1^ correspond to C═O of –COOH functional groups. Compared to 3‐GuA, the C═O stretching vibration peaks of 3‐GuA·PbI_2_ slightly shift toward a larger wavelength number from 1666 to 1672 cm^−1^. Meanwhile, the N‐H stretching vibrations of 3‐GuA, appearing at wavenumbers from 2800 to 3300 cm^−1^, greatly weaken compared with 3‐GuA·PbI_2_ mixture, indicating the strong chemical interactions between 3‐GuA and PbI_2_, Obviously, 3‐GuA, with both guanidine groups and ‐COOH functional groups, can form effective hydrogen & coordination bonds with perovskite precursor,^[^ ^10^ ^]^ which not only eliminate defects but also manage the crystallization of perovskite crystals, as shown in Figure 1c.

Having determined the interactions between 3‐GuA and PbI_2_, we processed to explore the interaction between 3‐GuA and perovskite precursor in solution environment. As shown in the FT‐IR spectra of Figure 1d, the S═O stretching vibration peaks of DMSO, appearing at wavenumber of 1040 cm^−1^ and 1017 cm^−1^, shift to a smaller wavenumber after incorporating PbI_2_, suggesting the molecular bonding between DMSO and PbI_2_.^[^ ^9^, ^11^ ^]^ In comparison, the S═O peaks of 3‐GuA· PbI_2_· DMSO move back to the large wavenumber from 1000 to 1013 cm^−1^, indicating a stronger interaction of 3‐GuA·PbI_2_. This strong molecular interaction of 3‐GuA·PbI_2_ is profited to reduce PbI_2_·DMSO phase after the first‐step low‐temperature annealing process and promotes the formation of a porous PbI_2_ layer. As proved by the X‐ray diffraction (XRD) measurements of Figure 1e, the intensity of XRD peak at ≈9.6° representing the formed PbI_2_·DMSO complex inside of the PbI_2_ film,^[^ ^12^ ^]^ slightly decreases after incorporating 3‐GuA into perovskite precursor, which corresponds to the reduced formation of PbI_2_·DMSO complex.

**Figure 2:**
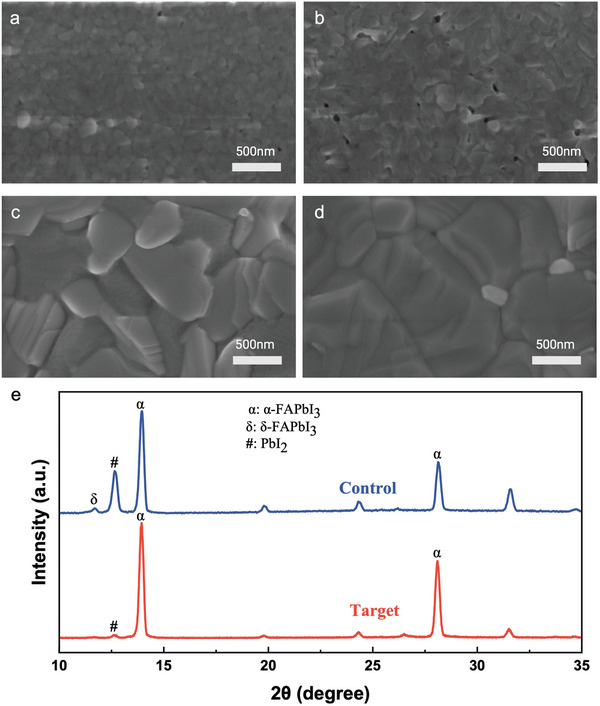
Morphology and film characteristics. SEM images of the PbI_2_ films without a) and with b) 3‐GuA. SEM image of the FAPbI_3_ film without c) and with d) 3‐GuA. e) XRD patterns of the control and target perovskite films after annealing.

**Figure 3:**
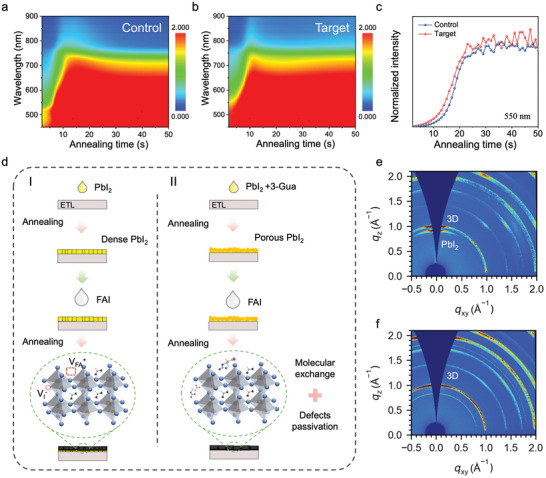
Crystallization of perovskite films. Time‐resolved in situ UV‐vis absorption spectra of the control a) and target b) perovskite thin films during the thermal annealing process. c) Dynamical evolution of absorption intensity of perovskite films at 550 nm during thermal annealing. d) schematic diagram of the film formation. 2D GWIAXS patterns of the control e) and target f) perovskite films.

The surface topography measurements intuitively revealed the influence of 3‐GuA on perovskite films. Scanning electron microscopy (SEM) and atomic force microscopy (AFM) images of PbI_2_ films without and with 3‐GuA incorporation were first captured to investigate the influence of 3‐GuA on PbI_2_ films. As demonstrated in **Figure** **2**a,b and 3‐GuA incorporated PbI_2_ films display a rougher surface with increased number of pinholes. From the AFM characterization (Figure S10, Supporting Information), the roughness (R_a_) increased from 4.99 to 5.56 compared to the pristine film. This porous morphology of the PbI_2_ films is beneficial for the ionic exchange in the second step of the crystallization process and promotes the formation of perovskite crystals.^[^ ^13^ ^]^ Therefore, compared with the pristine perovskite films, the SEM images of the 3‐GuA incorporated perovskite show an increased grain size with the decreased R_a_ from 27.7 nm to 24.2 nm (Figure 2c,d). The formation of this densely packed uniform grains is attributed to the strong ‐CO‐I and ‐NH‐I bonds between perovskite grains and 3‐GuA molecules during crystallization process.^[^ ^14^ ^]^ The increased crystal size with smoother surface is benefit to decrease boundary defects and improve interfacial contact,^[^ ^15^ ^]^ thus improving PSCs performance.

To elucidate the crystallinity of the perovskite films, X‐ray diffraction (XRD) measurements were conducted. As depicted in Figure 2e, the strong peaks observed at 14.3° and 28.4° correspond to the (001) and (002) planes of 3D α‐phase FAPbI_3_ perovskite crystals. Notably, the intensity of the PbI_2_ peaks at 12.7° is significantly reduced in the target perovskite film. This reduction suggests that 3‐GuA interacts with uncoordinated Pb^2+^ ions, thereby modulating film growth to achieve fully developed grains.^[^ ^10^, ^16^ ^]^ Consequently, higher quality perovskite films are obtained, as evidenced by the increased intensity of the characteristic peak in the XRD pattern (Figure 2e).

We further investigated the crystallization kinetics of the samples via time‐resolved in‐situ UV‐vis absorption measurement to explore how 3‐GuA influences the perovskite crystallization during the film fabrication. Figure S1 (Supporting Information) shows the in‐situ UV absorption spectra of PbI_2_ films during annealing process. As expected, the target PbI_2_ films tend to solidify faster, which is attributed to the reduced PbI_2_·DMSO interaction.^[^ ^16^ ^]^ The conversion processes of PbI_2_ to perovskite samples during thermal annealing treatment were tracked and recorded in **Figure** **3**a,b. The absorption curves of the fresh perovskite films red shift from the beginning stage of the annealing process and tend to stabilize after completing conversion of perovskite. Compared to the pristine samples, an accelerated red shift of absorption curves was observed for the target one. Meanwhile, we found that the absorption curves stabilize faster compared to the control one. This difference can be attributed to the accelerated perovskite conversion process of the film due to the more efficient molecular exchange of FA^+^ and 3‐GuA.^[^ ^17^ ^]^ To clearly illustrate our observation, the dynamic evolution of the absorption intensity at 550 nm as a function of time was plotted in Figure 3c. Obviously, the target films complete the solidification and conversion of perovskite faster. Therefore, the incorporation of 3‐GuA facilitates perovskite formation, resulting in accelerated perovskite crystallization kinetics.^[^ ^18^ ^]^

To better understand the effect of 3‐GuA on crystallization, a schematic diagram is demonstrated in Figure 3d. The addition of 3‐GuA reduces the formation of PbI_2_·DMSO adducts and forms new PbI_2_·3‐GuA intermediate adducts, which accelerates solvent evaporation and forms porous PbI_2_ films. Next, an intermodular exchange between 3‐GuA and FA^+^ promotes the crystallization of perovskite films. The formation of the high‐quality perovskite crystals was further proved by grazing‐incidence wide‐angle X‐ray scattering (GIWAXs) measurements (Figure 3e,f). The control films exhibit an out‐of‐plane diffraction ring at ≈0.9 Å^−1^, which can be attributing to the PbI_2_ residues.^[^ ^19^ ^]^ Generally, the preferential parallel orientation of the PbI_2_ fragments with respect to the 3D perovskite layer is unfavorable for stability and also triggers the hysteresis effect.^[^ ^7^, ^20^ ^]^ By comparation, the addition of 3‐GuA diminishes the scattering ring from PbI_2_ and contributes to the formation of high‐quality crystals. To further verify the optimized film quality, the grazing incidence X‐ray diffraction (GIXRD) was measured and shown in Figure S2a–c (Supporting Information). Obviously, the diffraction peaks gradually shift to the lower angle direction as the tilt angle increases from 0° to 60°, indicating the existence of out‐of‐plane residual stress. Compared to the control films, the XRD d_100_ versus sin^2^(ψ) plots show a lower positive slope for the control films compared to the control one, suggesting a decrease in the out‐of‐plane residual stress.^[^ ^21^ ^]^ Table S1 (Supporting Information) shows the calculated residual stress value. These results suggest that the molecular exchange dominated crystallization process improves the crystal quality and reduces the residual stress of perovskite films, which are beneficial to the intrinsic stability of perovskite crystals bulks.

**Figure 4:**
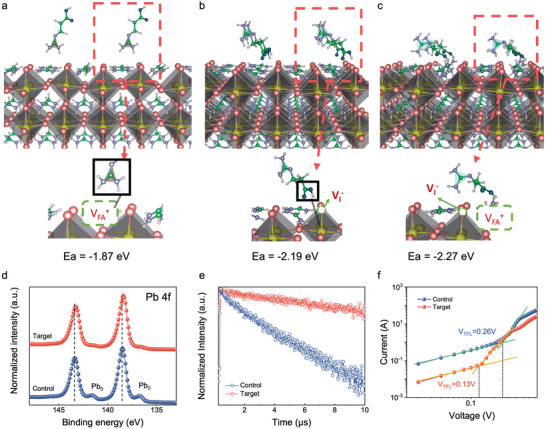
Passivation effects of 3‐GuA. a‐c) Adsorption configurations of 3‐GuA on perovskite crystals and their corresponding ΔE_ads_. d) Pb 4f spectra of control and target FAPbI_3_ perovskite films. e) TRPL spectra of perovskite films. e) Electron‐only devices of PSCs with the structure of ITO/SnO_2_/Perovskite/PCBM/Ag.

Having assured about the role of 3‐GuA on crystallization kinetics, we processed to investigate their effect on perovskite crystals. Density functional theory (DFT) calculations indicate that 3‐GuA preferentially bounds to perovskite surface/boundary and thus passivates the perovskite surface defects. Additionally, the multifunctional groups of 3‐GuA molecules can bond with under‐coordinated lead and halide ions, thus enabling dual passivation of both lead and halide defects (**Figure** **4**a–c).

X‐ray photoelectron spectroscopy (XPS) was conducted to examine the chemical interactions of 3‐GuA with perovskite. As shown in Figure S3b (Supporting Information), the Pb 4f peaks of PbI_2_·3‐GuA films shift to lower binding energy compared with PbI_2_ films, suggesting strong interaction of PbI_2_ with 3‐GuA. Meanwhile, the Pb 4f peaks (Figure 4d) of perovskite films also shift to lower binding energy after incorporating 3‐GuA, indicating the strong bonding of 3‐GuA on perovskite crystals. Additionally, two obvious peaks, related to Pb^0^ of uncoordinated Pb in perovskite films, can be seen at 141.6 eV and 136.7 eV for control films. Generally, Pb^0^ trap states act as recombination center of charge carriers, leading to increased non‐radiative recombination and a poor stability of PSCs.^[^ ^22^ ^]^ By comparation, the intensity of the Pb^0^ peaks is significantly suppressed, suggesting the removed uncoordinated Pb in target perovskite films.

To study the passivation effect of 3‐GuA in perovskite films, the steady‐state photoluminescence (PL) and time‐resolved photoluminescence (TRPL) measurements were performed. As confirmed by PL spectra in Figure S5 (Supporting Information), 3‐GuA does indicate passivation effect and results in higher PL than the control film. Furthermore, we fitted the TRPL curves via a bi‐exponential decay and calculated the lifetime. As shown in Figure 4e and Table S2 (Supporting Information), the average decay time increases from 0.3 µs (Control) to 1.6µs (Target), indicating an excellent defect passivation effect of 3‐GuA on FAPbI_3_ perovskite surface. To quantitatively characterize the effects of 3‐GuA on the defect passivation of perovskite films, we performed the space‐charge limited current (SCLC) with the electron‐only device in the structure of ITO/SnO_2_/Perovskite/PCBM/Ag (Figure 4f). The V_TFL_ of the control and target devices are 0.26 and 0.13 V, respectively. The calculated trap density N_t_ for target devices is 7.6×1015 cm^−3^, which a about one order of magnitude lower than that of the control one (1.53×1016 cm^−3^). The decreased defect density indicates that the incorporation of 3‐GuA enhances crystal quality and effectively passivates the perovskite defects.

**Figure 5:**
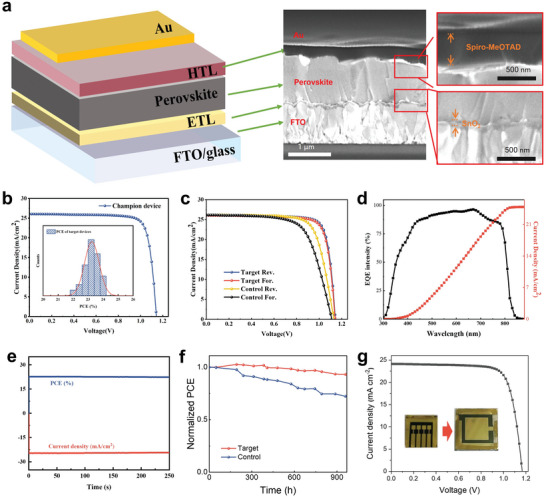
Device structure and performances. a) Device architecture and the cross‐section SEM of PSC. b) *J‐V* curves of the champion device. Inset: Statistical histogram of target devices. c) *J‐V* curves of PSCs from forward and reverse scans. d) EQE and integrated current density curve of a target device. e) Steady‐state photocurrent and power output at the maximum power point for target device at 0.88 V. f) Aging test of the control and target device. g) *J‐V* curves of target device with 1cm^2^ active area.

PSCs were fabricated with the device configuration of fluorine‐doped tin oxide (FTO)/electron transporting layer (ETL)/perovskite layer (with/without 3‐GuA)/hole transporting layer (HTL)/Au. **Figure** **5**a presents the cross‐section SEM image of the device, in which the thickness of perovskite layer is ≈750± 20 nm. As shown in Figure 5b,c, the control device exhibits a *J_sc_* of 25.15 mA cm^−2^, a *V_OC_* of 1.13 V, and an FF of 73.58%, yielding a PCE of 20.92%. In comparison, the target PSCs deliver a *V_OC_* of 1.17 V, a *J_SC_* of 25.27 mA cm^−2^, and an improved FF of 81.68%, contributing to an elevated PCE of 24.17%. The detailed device parameters, including *V_OC_*, *J_SC_*, FF, and PCE, are summarized in Table S3 (Supporting Information). The optimization process of target devices is displayed in Figure S6 (Supporting Information), and the device parameters are summarized in Table S4 (Supporting Information). The statistical PCE histogram of the target devices is inserted in Figure 5b, which suggests a high reproducibility of high‐efficiency PSCs.

Figure 5c and Table S5 (Supporting Information) plot the *J‐V* curves of PSCs measured in the reverse and forward scan modes. Compared with the control one, a negligible hysteresis was observed for the target PSCs. This reduced hysteresis benefits from the high‐quality perovskite crystals and effect passivation of 3‐GuA, corresponding to the reduced defect density and the suppressed carrier accumulation/recombination.^[^ ^23^ ^]^ The integrated photocurrent from the external quantum efficiency (EQE) spectrum also matches well with the values obtained from the *J‐V* measurements (Figure 5d; Figure S7, Supporting Information). We compared our device performance with reported pure FAPbI_3_‐based PSCs prepared under ambient air conditions. Table S6 (Supporting Information) shows our PSCs represent one of the most advanced devices. To ensure the credibility of the optimized PSCs characteristics, the stabilized power outputs were measured at the maximum power point (Figure 5e), giving a stabilized *J_sc_* of ≈25 mA cm^−2^ and a PCE of ≈23%. The optimized devices can be stabilized after operating more than 250s under AM1.5 illumination, indicating excellent stable/steady‐state output of our PSCs. Meanwhile, device exhibits excellent stability and maintains 93% of its initial PCE after 960 h in ambient air (Figure 5f), indicating excellent application potential of our PSCs. To further prove the reproducibility of high device performance, we achieved high‐efficiency PSCs of approaching 22% in a large area device 100 mm^2^ (Figure 5g).

## 3 Conclusion

In summary, we have demonstrated a straightforward dual functional strategy, assisted by 3‐GuA, to achieve air ambient fabricated FAPbI_3_ PSCs with excellent efficiency and stability. Systematic investigations indicate that 3‐GuA aids in managing excess PbI_2_ and facilitates the formation of high‐quality α‐FAPbl_3_. Moreover, 3‐GuA predominantly interacts with the grain boundaries and surfaces of the perovskite films, serving to passivate defects and stabilize the crystal structure. As a result, the champion PSC achieves an impressive efficiency of 24.2% and retains 93% of its initial efficiency after over 900 h in ambient environment (25 °C, 30% relative humidity). This opens a new avenue for the ambient fabrication of highly efficient and stable FAPbI_3_ PSCs.

## 4 Experimental Section

### Materials

Dimethylformamide (DMF, 99.8%, anhydrous), dimethyl sulfoxide (DMSO, 99.9%, anhydrous), chlorobenzene (CB, 99.8%, anhydrous), isopropanol (IPA, ≥99.5%), lead iodide (PbI_2_, 99.99%), bis(trifluoromethane)sulfonimide lithium salt (Li‐TFSI), acetonitrile (ACN), 4‐tert‐butylpyridine (tBP) and n‐Butylammonium bromide (BABr, ≥98%) were purchased from Sigma Aldrich. 3‐Guanidinopropionic acid (3‐GuA, ≥99%) was purchased from Aladdin. Formamidinium iodide (FAI, ≥99%), methylammonium chloride (MACl, ≥98%), N^2^, N^2^, N^2′^, N^2′^, N^7^, N^7^, N^7′^, N^7′^‐octakis(4‐methoxyphenyl)‐9,9′‐spirobi[9H‐fluorene]‐2,2′,7,7′‐tetramine (Spiro‐MeOTAD, 99%) were purchased from Xi'an Polymer Light Technology in China. All the materials are stored in the nitrogen filled glove box to avoid the water.

### Device Fabrication

Substrates (FTO/Glass) were cleaned by sonication in detergent water solution for 20 min and then sequentially ultrasonicate in deionized water, acetone, and isopropanol each for 20 min. After drying under N_2_ flow, the substrates were further cleaned with UV ozone treatment for 15 min before use. Then, ≈10 nm SnO_2_ compact layer was deposited on FTO via chemical bath deposition (CBD). This solution process requires the substrate to be vertically placed in the solution with a holder and heated in an oven at 90 °C for 4.5 h. The CBD solution was prepared by mixing 2.50 g of Urea (>99.5%), 550 mg of SnCl_2_⋅2H_2_O (>99.99%), 2.50 mL of Hydrochloric acid (HCl, 37%), and 50 µL of Thioglycolic acid (TGA, ≥99%), per 200 mL of deionized water. After depositing, the substrates were sequentially ultrasonicate in deionized water and isopropanol each for 5 min. Then further annealed at 170 °C for 60 min. The substrates were left to cool down to room temperature after annealing, then cleaned with ultraviolet ozone for 15 min to improve surface wettability. The perovskite layer was carefully deposited within an air‐dry box, where conditions were meticulously regulated to maintain a relative humidity of 30–40% and a room temperature of 25 °C. Spin‐coating of 1.5 M PbI_2_ in DMF: DMSO (9:1) solvent onto SnO_2_/FTO at 1800 rpm for 30 s and annealing at 70 °C for 1 min, followed by cooling to room temperature was carried out. To compare the results, a 1 mg mL^−1^ concentration of 3‐GuA was added to the PbI_2_ solution. For perovskite film deposition, a solution of FAI: MACl (90mg: 18 mg in 1 mL IPA) was spin‐coated onto the PbI_2_ at a spin rate of 2000 rpm followed by annealing at 150 °C for 15 minutes. After perovskite formation, the samples were transferred to a nitrogen‐filled glove box for further processing, primarily to facilitate the thermal evaporation of the gold electrode. The passivation layer was then applied by spin‐coating the BABr dissolved in IPA (2 mg mL^−1^) at a spin rate of 5000 rpm and annealing at 100 °C for 5 min. The hole transport layer was then deposited on top of the passivation layer at a spin rate of 3500 rpm for 30s using a Spiro‐OMeTAD solution consisting of 72.3 mg Spiro‐OMeTAD powder, 17.5 µL Li‐TFSI stock solution (260 mg Li‐TFSI in 1 mL acetonitrile), 29 µL tBP, and 1 mL CB. Finally, 80 nm Au film was thermally evaporated as the top electrode using a mask with 0.04 cm^2^ active area. Additionally, the subsequent processes after perovskite formation could also similarly execute in ambient air at room temperature, ensuring uniformity with the conditions established for the perovskite layer. The only exception to this procedure is the deposition of the gold electrode, which is carried out in a glovebox within a vacuum chamber to ensure optimal quality and adherence.

### Film and Device Characterizations

FTIR spectra were collected by the Thermo Scientific NICOLET iS50 FT‐IR spectrometer. Crystalline structure was explored on a Rigaku SmartLab X‐ray diffractometer with Cu Kα radiation in a step of 0.01° and θ‐2θ scan mode from 5° to 50°. Film morphology of the perovskite was measured using a high‐resolution field emission SEM (TESCAN VEGA3). The surface roughness and potential were tested using a MultiMode 8‐HR Atomic Force Microscope (AFM, Bruker) with a tapping amplitude modulation mode. The in‐situ UV–vis absorption spectra were measured using an F20‐UVX spectrometer (Filmetrics, Inc.) equipped with tungsten halogen and deuterium light sources (Filmetrics, Inc.). Steady‐state PL and TRPL transient decay spectra were measured using a PL spectrometer (Edinburgh Instruments, FLS920) with the excitation source of 636.2 nm picosecond pulsed diode laser (EPL‐635, ≈5 nJ cm^−2^) and detected at 780 nm. Photoelectron spectroscopy measurements were conducted by an electron analyzer (ESCALAB XI, Thermo Fisher Scientific) with a monochromatic X‐ray source (Al Kα, hν = 1486.7 eV) for XPS in an ultrahigh vacuum chamber. For the completed devices, *J*–*V* curves were obtained using a Keithley 2400 Source Meter under standard AM 1.5 G illumination (Enli Technology Co. Ltd., Taiwan), and the light intensity was calibrated by a standard KG‐5 Si diode. EQE spectra were measured with a QE‐R 3011 EQE system (Enli Technology Co. Ltd., Taiwan) using 210 Hz chopped monochromatic light ranging from 300 to 850 nm.

### DFT Calculations

The first principles computations were carried out by Vienna Ab initio Simulation Package (VASP) based on density functional theory. Generalized gradient approximation (GGA) with the Perdew‐Burke‐Ernzerhof (PBE) functional was used to describe the exchange‐correlation interaction. The projector augmented wave pseudopotentials were adopted to describe the ion‐electron with a cutoff energy of 500 eV. The K‐point grid of Brillouin zone was tested and sampled by 3×3×1 within Gamma‐Pack. The electronic energy and forces were converged to within 10^−5^ eV and 0.03 eV Å^−1^, respectively. The vacuum layer in the slab model is larger than 15 Å to avoid superficial interaction between periodical slabs.

## Conflict of Interest

The authors declare no conflict of interest.

## Supporting information



Supporting Information

## Data Availability

The data that support the findings of this study are available from the corresponding author upon reasonable request.
