# Supplementary material for: Tailoring Crystal Growth Regulation and Dual Passivation for Air‐Processed Efficient Perovskite Solar Cells
Source: Adv Sci (Weinh). 2025 Feb 19;12(14):2407401. doi: 10.1002/advs.202407401 (PMC11984867; doi:10.1002/advs.202407401)
Supplement: Supplementary file 1 — Supporting Information [file ADVS-12-2407401-s001.docx]

**Supplementary information**

Tailoring Crystal Growth Regulation and Dual Passivation for Air-Processed Efficient Perovskite Solar Cells

*Qianyi Li^1^, Dongyang Li^1^, Zhiqi Li^1*^, Qiong Liang^1,2^, Patrick W. K. Fong^1^, Yu Han^1^, Kuan Liu^1,2^, Jiangsheng Yu^1^, Peng Bai^1^, Tao Zhu^1^, Yang Bai^3,4^, Guang Yang^1^, Zhiwei Ren^1,2*^, and Gang Li^1,2*^*

Q. Li, D. Li, Dr. Z. Li, Q. Liang, P. Fong, Y. Han, Dr. K. Liu, Dr. J. Yu, P. Bai, Dr. T. Zhu, Dr. Y Bai, Dr. G. Yang, Dr. Z. Ren, and Prof. G. Li

^1^Department of Electrical and Electronic Engineering, Photonic Research Institute (PRI), Research Institute of Smart Energy (RISE), The Hong Kong Polytechnic University, Hung Hom, Kowloon, Hong Kong, China.

^2^Research Institute for Intelligent Wearable Systems (RI-WEAR), The Hong Kong Polytechnic University, Hung Hom, Kowloon, Hong Kong, China.

^3^Faculty of Materials Science and Energy Engineering, Shenzhen University of Advanced Technology, Shenzhen 518107, China.

^4^Institute of Technology for Carbon Neutrality, Shenzhen Institute of Advanced Technology, Chinese Academy of Sciences, Shenzhen 518055, China.

E-mail: zhiqili@polyu.edu.hk (Z. L.); [zhiweipv.ren@polyu.edu.hk](mailto:zhiweipv.ren@polyu.edu.hk) (Z. R.); [gang.w.li@polyu.edu.hk](mailto:gang.w.li@polyu.edu.hk) (G. L.)

**a**

**b**

**Figure S1**. In-situ UV absorption spectra of control (a) and target (b) PbI_2_ films during annealing process.

**Figure S2.** GIXRD spectra of control (a) and target (b) perovskite films at tilt angle Φ=90°. And XRD 2θ versus sin2ψ plots for control and target films at Φ=90° (c).

**a**

**b**

**c**

**
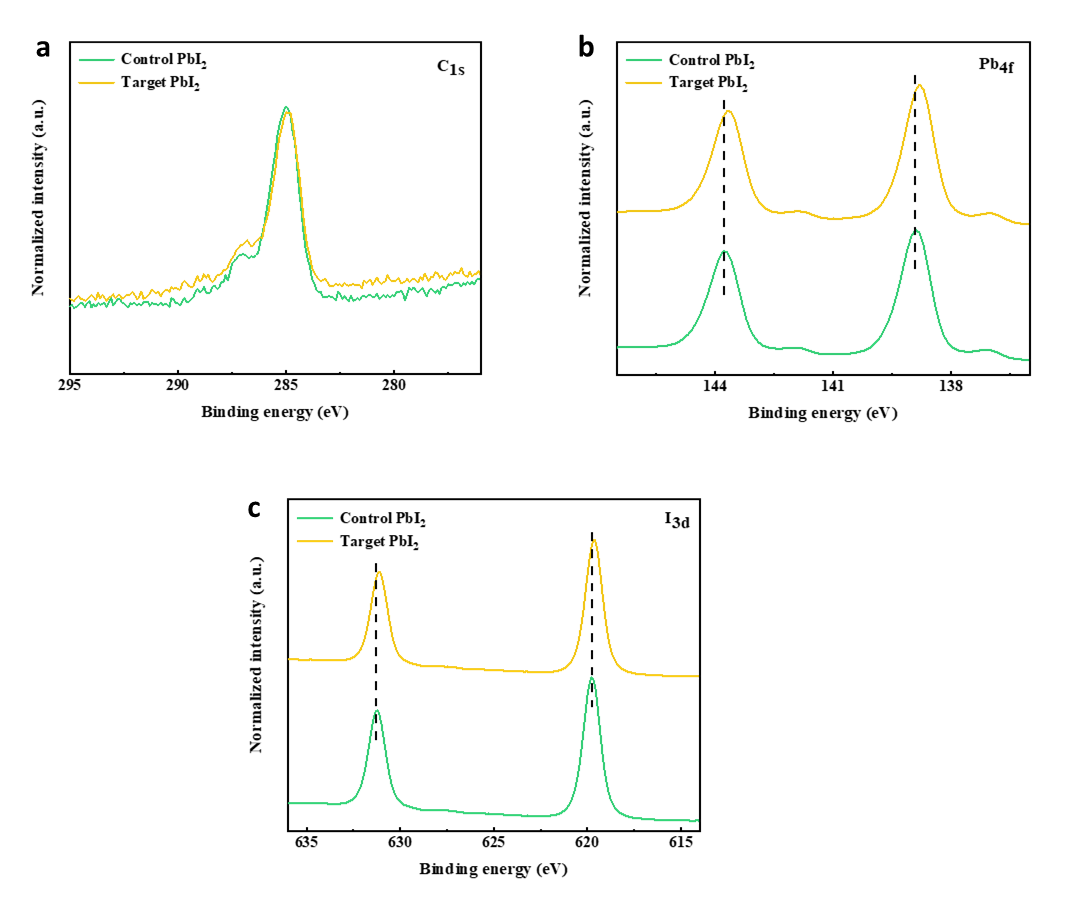
Figure S3**. XPS spectra of PbI_2_ films. a) C 1s spectra of control and target PbI_2_ films. b) Pb 4f spectra of control and target PbI_2_ films. c) I 3d spectra of control and target PbI_2_ films.

**
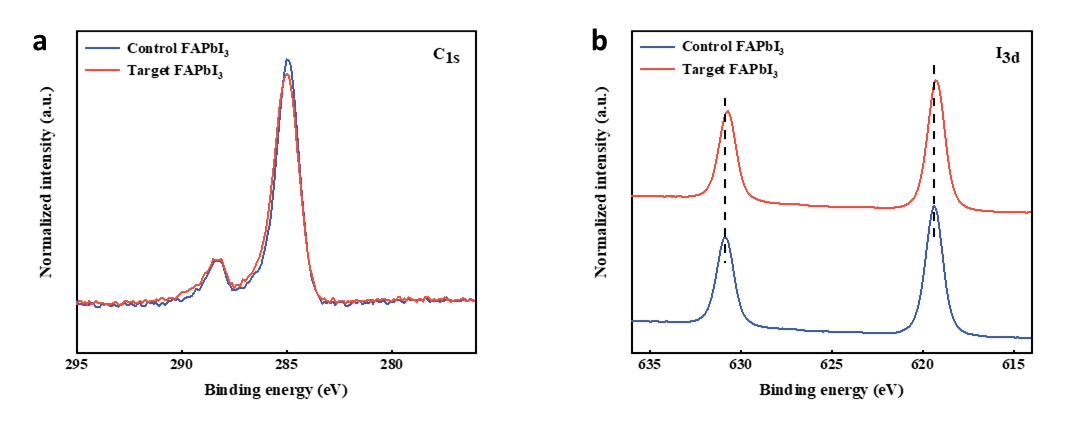
Figure S4.** XPS spectra of perovskite films. a) C 1s spectra of control and target FAPbI_3_ perovskite films. b) I 3d spectra of control and target FAPbI_3_ films.

**Figure S5.** Steady-state PL spectra of perovskite films.

**Figure S6.** EQE and Differential of EQE of perovskite solar cells. The corresponding optical bandgap of the target perovskite was 1240 / 809=1.53 eV.


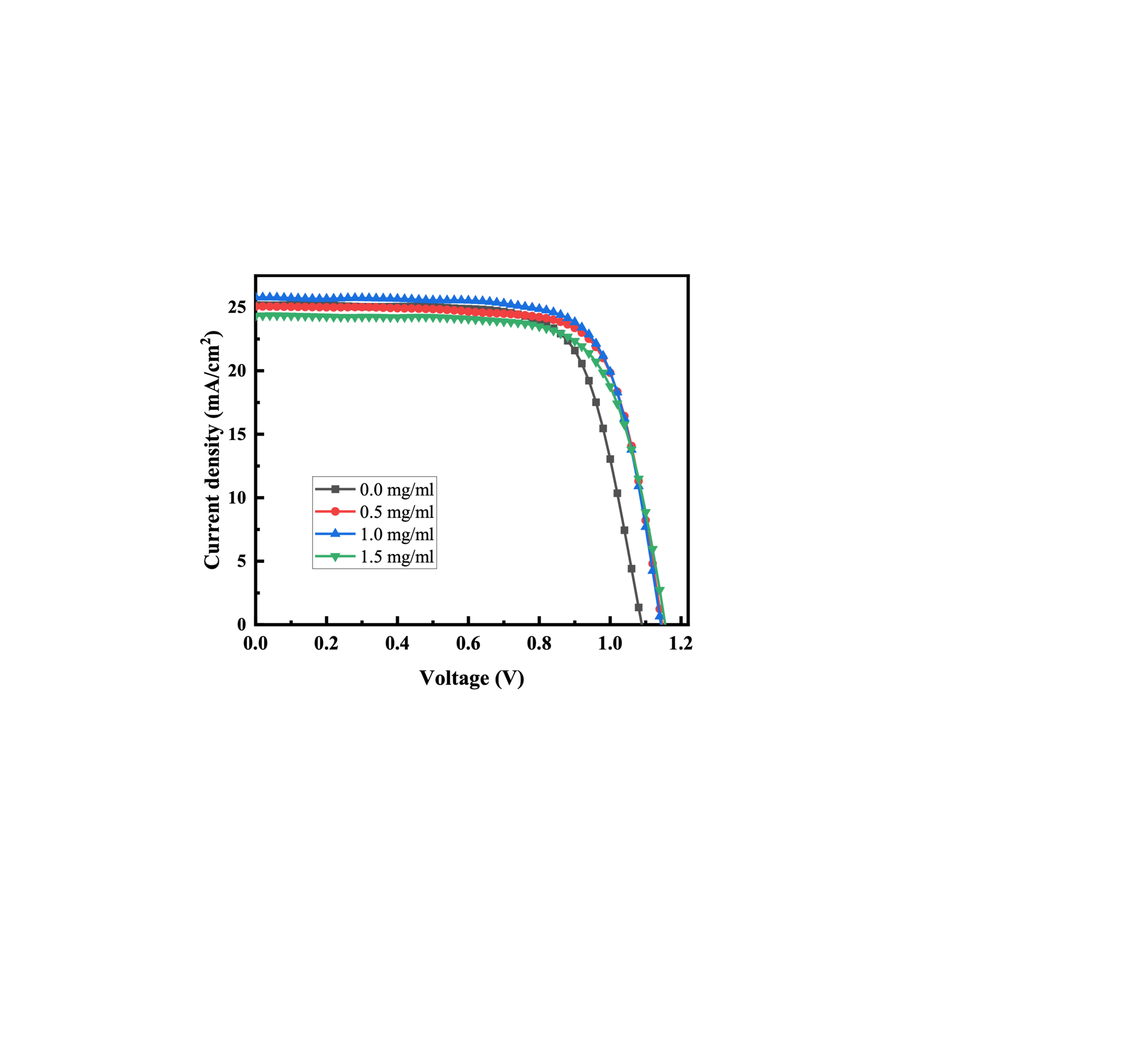


**Figure S7.** The J-V curve results of additive (3-GuA) concentration gradient test.


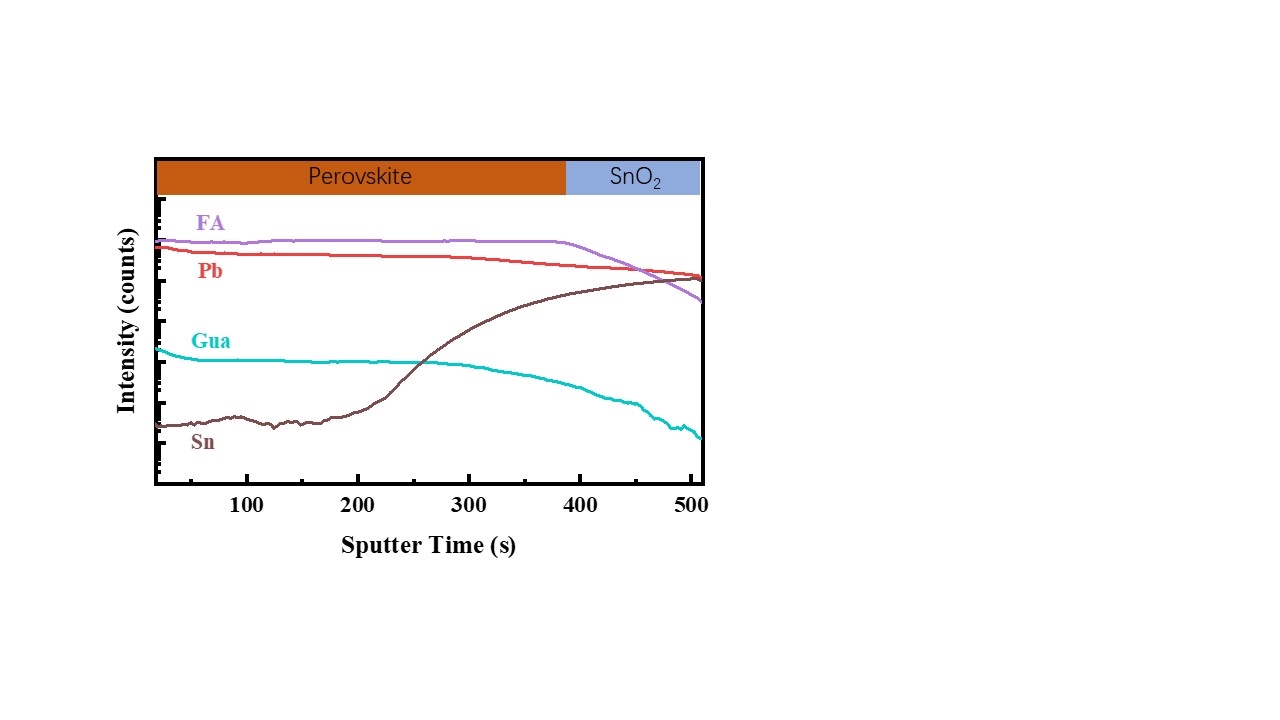


**Figure S8**. ToF-SIMS depth profiles of 3-GuA-based perovskite film/SnO_2_ substrate.


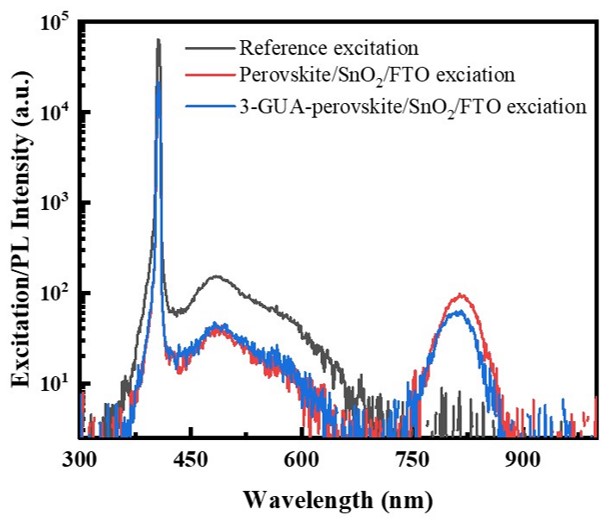


Figure S9. Photoluminescence quantum yield (PLQY) of the control and target FAPbI_3_ perovskite films.


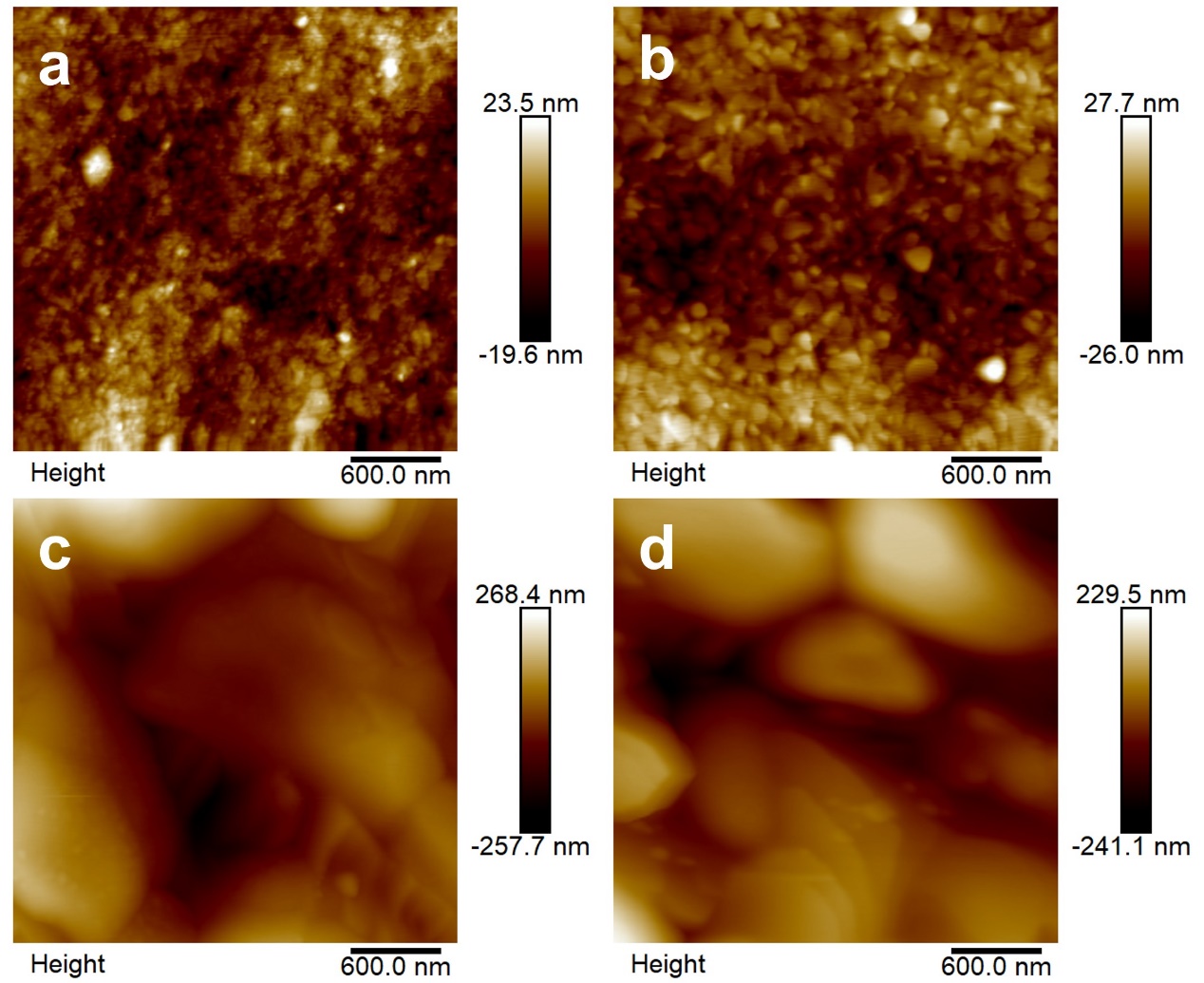


Figure S10. Atomic force microscopy (AFM) images of pristine PbI_2_ film (a), the additive PbI_2_ film (b), the pristine FAPbI_3_ film (c) and the additive FAPbI_3_ film (d).


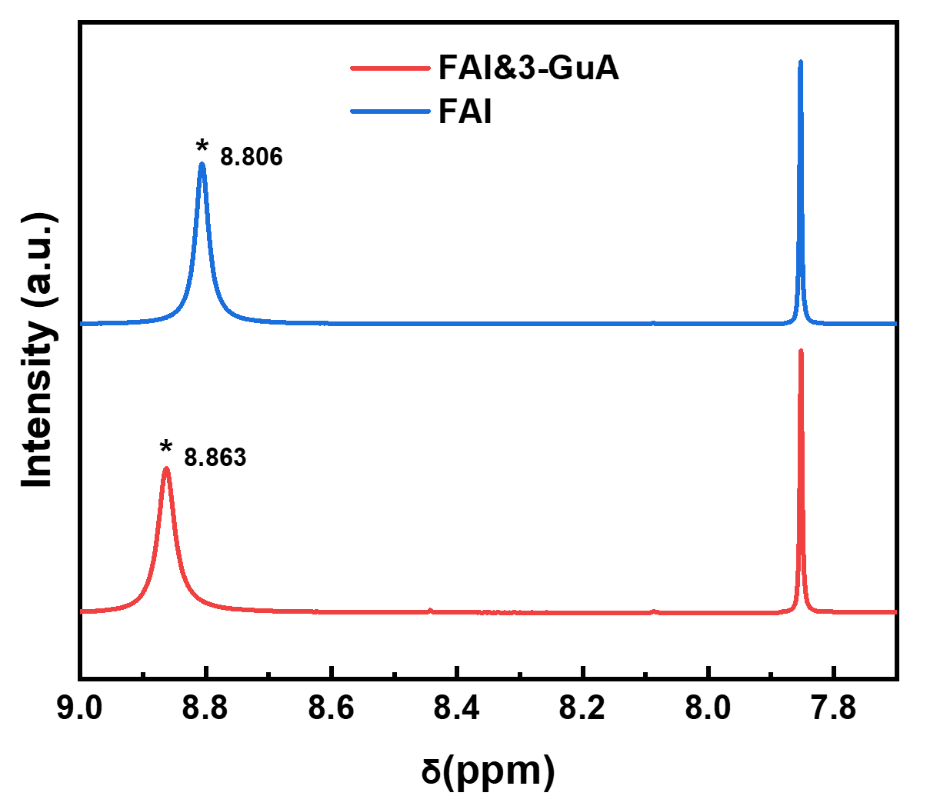
Figure S11. The ^1^H NMR spectra of FAI, and the mixture of FAI and 3-GuA.


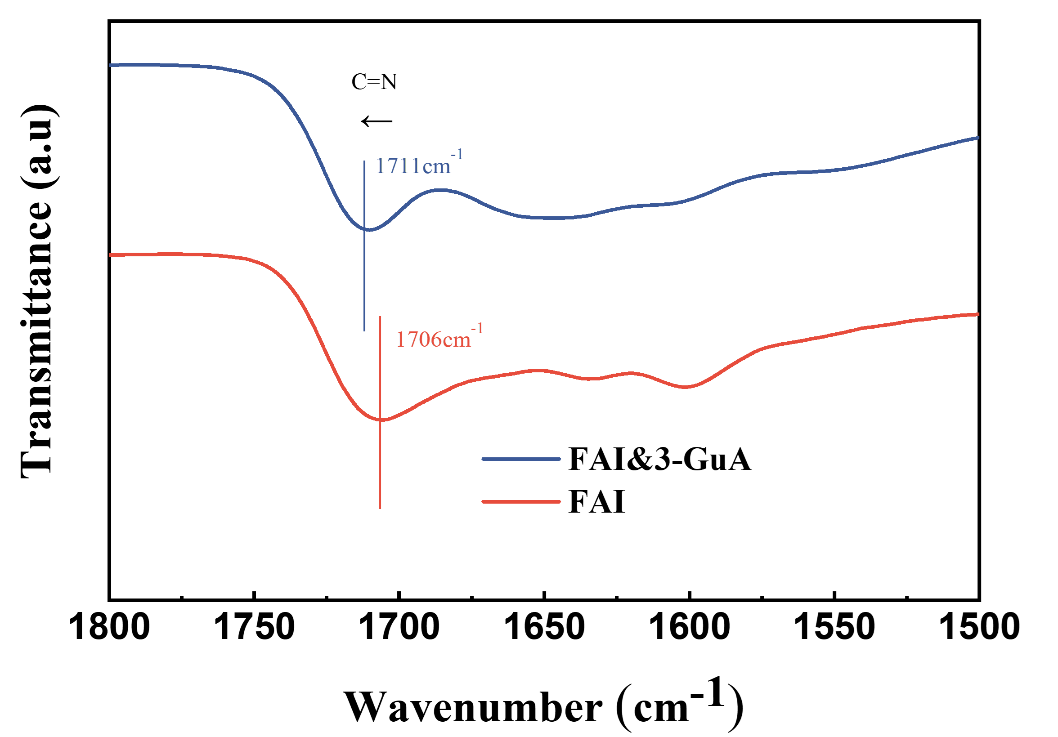


Figure S12. Fourier transform infrared (FT-IR) spectra of FAI and FAI & 3-GuA.


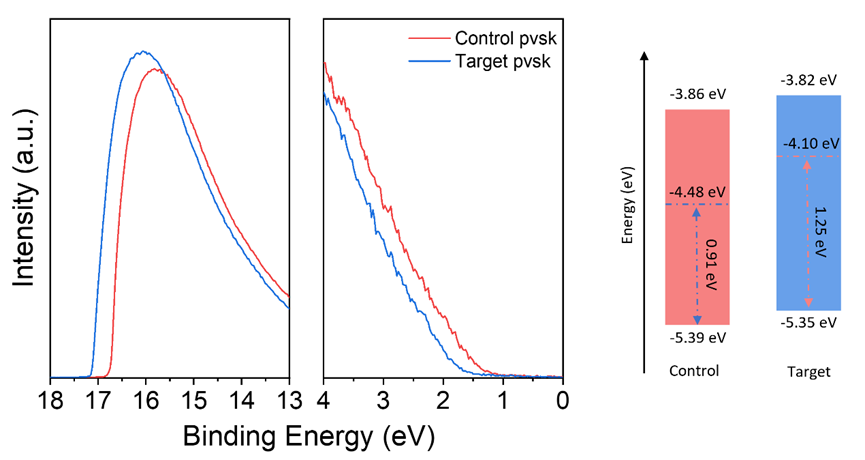


Figure S13. Energy band of the control (without 3-GuA treatment) and target (with 3-GuA treatment) perovskite.

|  | **Control** | **Target** |
| --- | --- | --- |
| σvm, MPa: | 12.31 | 5.12 |

**Table S1.** Calculated residual stress value.

|  | *τ_1_* [ns] | *τ_2_* [ns] | *A_1_* | *A_2_* | *τ_ave_* [us] |
| --- | --- | --- | --- | --- | --- |
| **Control** | 34.67 | 283.38 | 103.63 | 377.62 | 0.3 |
| **Target** | 6.27 | 1605.28 | 53.65 | 492.23 | 1.6 |

**Table S2.** Fitted data of the TRPL based on the control and target perovskite films.

|  | **Voc** | **Jsc** | **FF** | **PCE** |
| --- | --- | --- | --- | --- |
| Control device | 1.13 | 25.15 | 73.58 | 20.92% |
| Target device | 1.17 | 25.27 | 81.68 | 24.17% |

**Table S3.** Performance of 0.04cm^2^ devices.

|  | **Voc** | **Jsc** | **FF** | **PCE** |
| --- | --- | --- | --- | --- |
| Forward scan | 1.158 | 24.92 | 72.89 | 21.03% |
| Reverse scan | 1.163 | 24.88 | 75.96 | 21.99% |

**Table S4.** Performance of a 1cm^2^ device.

| **3-GuA** Dope concentration | **Voc** | **Jsc** | **FF (%)** | **PCE (%)** |
| --- | --- | --- | --- | --- |
| 0 mg/mL | 1.093 | 25.03 | 72.07 | 19.72 |
| 0.5 mg/mL | 1.154 | 25.06 | 77.10 | 22.29 |
| **1 mg/mL** | **1.158** | **25.93** | **77.76** | **23.34** |
| 1.5 mg/mL | 1.161 | 24.76 | 74.82 | 21.51 |

**Table S5.** The results of additive (3-GuA) concentration gradient test.

| Fabrication condition | Active layer | PCE (%) | Year of publication | reference |
| --- | --- | --- | --- | --- |
| Ambient air fabrication | FAPbI_3_ | 20.19 | 2022 | [1] |
|  |  | 21.6 | 2022 | [2] |
|  |  | 21.17 | 2024 | [3] |
|  |  | 23.3 | 2024 | [4] |
|  |  | 19.9 | 2024 | [5] |
|  |  | 23.75 | 2024 | [6] |
|  |  | **24.17** | **/** | **This work** |

**Table S6**. The performance comparison of pure FAPbI3 perovskite films prepared under ambient air conditions.

[1] D. Lin, Y. Gao, T. Zhang, Z. Zhan, N. Pang, Z. Wu, K. Chen, T. Shi, Z. Pan, P. Liu, W. Xie, *Adv. Funct. Mater.* **2022**, 32, 2208392.

[2] T. Du, T. J. Macdonald, R. X. Yang, M. Li, Z. Jiang, L. Mohan, W. Xu, Z. Su, X. Gao, R. Whiteley, C.-T. Lin, G. Min, S. A. Haque, J. R. Durrant, K. A. Persson, M. A. McLachlan, J. Briscoe, *Adv. Mater.* **2022**, 34, 2107850.

[3] M. K. A., Mohammed, M. I., Abualsayed, B. J., Mohammed, et al. *ACS Appl. Energy Mate.* **2024**, 7, 3, 1358–1368.

[4] B., Guo, X., Chen, H., Luo, G. O., Odunmbaku, T., Jiang, et al. *Solar RRL.* **2024**, 8, 2300934

[5] Hytham Elbohy, Hiroo Suzuki, Takeshi Nishikawa, Aung Ko Ko Kyaw, and Yasuhiko Hayashi. *ACS Applied Energy Materials* **2024** 7 (7), 2925-2937

[6] G Luo, L Zhang, L Guo, X Geng, P Ren, Y Zhang. *Journal of Energy Chemistry*, **2024**.03.014, 625-634
